# Gastric antral vascular ectasia in systemic sclerosis: a study of its epidemiology, disease characteristics and impact on survival

**DOI:** 10.1186/s13075-022-02790-1

**Published:** 2022-05-10

**Authors:** Kathleen Morrisroe, Dylan Hansen, Wendy Stevens, Joanne Sahhar, Gene-Siew Ngian, Catherine Hill, Janet Roddy, Jennifer Walker, Susanna Proudman, Mandana Nikpour

**Affiliations:** 1 Department of Medicine, The University of Melbourne at St Vincent’s Hospital (Melbourne), 41 Victoria Parade, Fitzroy, Victoria 3065 Australia; 2 Department of Rheumatology, St Vincent’s Hospital (Melbourne), 41 Victoria Parade, Fitzroy, Victoria 3065 Australia; 3 Department of Medicine, Monash University, Clayton and Monash Health, 246 Clayton Road, Clayton, Victoria 3168 Australia; 4 Rheumatology Unit, Royal Adelaide Hospital, North Terrace, Adelaide, SA 5000 Australia; 5 Rheumatology Unit, The Queen Elizabeth Hospital, Woodville Road, Woodville, SA 5011 Australia; 6 Discipline of Medicine, University of Adelaide, Adelaide, SA 5000 Australia; 7 Department of Rheumatology, Royal Perth Hospital, Perth, Australia; 8 Rheumatology Unit, Flinders Medical Centre (Adelaide), Flinders Drive, Bedford Park, South Australia 5042 Australia

**Keywords:** Systemic sclerosis, Scleroderma, Gastric antral vascular ectasia

## Abstract

**Background:**

To describe the epidemiology, determinants and survival impact of gastric antral vascular ectasia (GAVE) in systemic sclerosis (SSc).

**Methods:**

Consecutive SSc patients prospectively enrolled in the Australian Scleroderma Cohort Study (ASCS) were included. Univariable and multivariable logistic regression were used to determine the associations of GAVE with clinical manifestations and serological parameters. Kaplan-Meier (K-M) survival curves were used to estimate survival.

**Results:**

The prevalence of GAVE in this SSc cohort of 2039 SSc patients was 10.6% (*n* = 216) over a median follow-up period of 4.3(1.7–8.4) years. SSc patients with a history of GAVE compared with those without a history of GAVE were older at SSc onset [49.5 (40.0–58.2) vs 46.7 (36.0–56.7) years, *p* = 0.05]; more likely to have diffuse disease subtype (dcSSc) (35.3% vs 24.1%, *p* < 0.001); be negative for Scl-70, U1RNP and Scl/PM antibody (4.0% vs 16.1%, *p* < 0.001, 3.5% vs 7.4%, *p* = 0.041, 0.0% vs 2.0%, *p* = 0.042; and respectively) and positive for RNAP III antibody (24.9% vs 8.3%, *p* < 0.001). Those with GAVE had a worse HRQoL (*p* = 0.002). Independent determinants of GAVE included the presence of RNAP III antibody (OR 3.46, *p* < 0.001), absence of Scl-70 antibody (OR 0.23, *p* = 0.001), presence of GIT dysmotility (OR 1.64, *p* = 0.004), and digital ulcers; pits; or digital amputation (OR 1.59, *p* = 0.014).

**Conclusions:**

GAVE is an underestimated and underappreciated SSc manifestation of SSc, which occurs with a relatively high frequency. Identifying an at-risk GAVE phenotype, as presented herein, is of practical importance as screening may prove advantageous given GAVE can be easily diagnosed and treated.

## Key messages

1. Gastric antral vascular ectasia is an underestimated and underappreciated clinical manifestation of systemic sclerosis.
2. Identifying an at-risk GAVE phenotype is of practical importance as it can easily be diagnosed and treated.
3. Iron studies, performed on a six-monthly basis, are a simple cost-effective screening tool for gastric antral vascular ectasia.

## Introduction

Australia has one of the highest reported prevalences of systemic sclerosis (SSc), an autoimmune connective tissue disease characterized by vasculopathy and fibrosis [1]. SSc is arguably the most devastating of the rheumatological diseases, irreparably damaging multiple organs and shortening life expectancy by two decades [1]. The clinical manifestations of SSc are multi-organ and diverse with vascular manifestations, namely cardiopulmonary and renal involvement, contributing to its high mortality, and gastrointestinal tract (GIT) involvement leading to its high morbidity and poor health-related quality of life (HRQoL) [2–4]. Whilst there has been extensive research dedicated to SSc-related vascular manifestations, including pulmonary arterial hypertension (PAH) and SSc renal crisis (SRC), little attention has been focused on gastric antral vascular ectasia (GAVE), which is an under-recognised yet treatable SSc vascular and gastric manifestation.

**Fig. 1:**
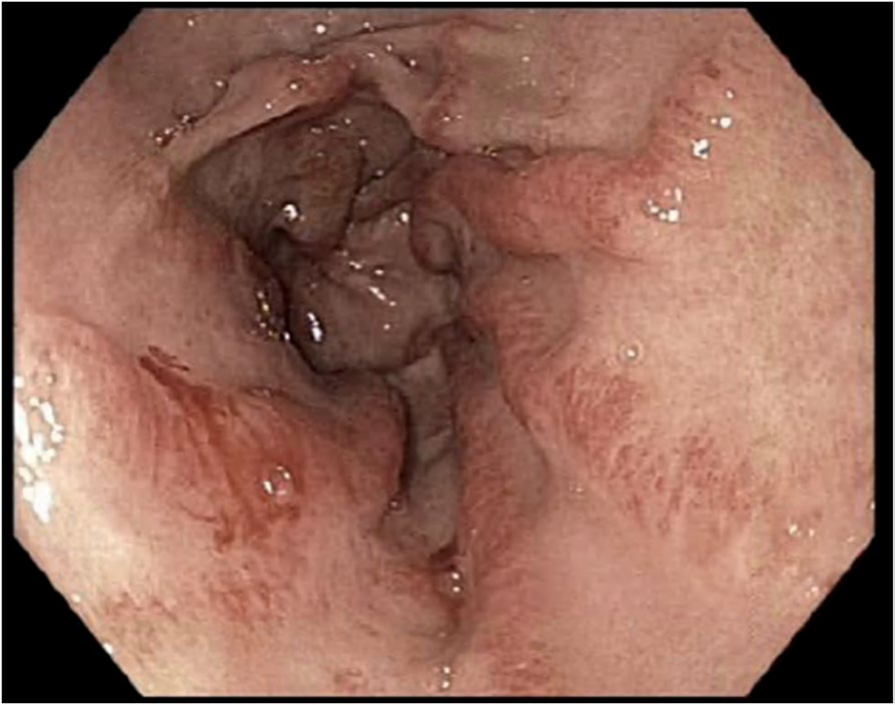
Gastric antral vascular ectasia on endoscopy. This endoscopic image shows gastric antral vascular ectasia as prominent, flat or raised erythematous stripes, radiating from the antrum to the pylorus

GAVE was first described endoscopically in 1953 by Rieder et al. [5] as an “erosive type of gastritis with marked veno-capilliary ectasia” in a patient presenting with chronic iron deficiency anaemia. In 1984, it was more accurately described as “longitudinal antral folds … converging on the pylorus, containing visible columns of tortuous red ectatic vessels” [6], features which are now considered pathognomonic for a diagnosis of GAVE [7]. The exact pathogenesis of GAVE, as with many other SSc manifestations, remains unknown [7]. Histological features typically seen in GAVE include the presence of hyperplasia of the mucosa with capillary ectasia and thrombosis, fibromuscular hyperplasia of the lamina propria and abnormal vessels in the submucosa [8, 9]. These endoscopic appearances resemble the stripes on a watermelon, hence the term “watermelon stomach” [8]. Figure 1a shows an endoscopic appearance of GAVE. These histological changes are not too dissimilar to the histological changes of inflammation, proliferation and thrombus formation seen in other SSc vascular manifestations, such as PAH and SRC [10, 11], leading to speculation that GAVE is a purely vascular rather than a specific gastric manifestation of SSc. Figure 1b shows a histological appearance of GAVE.

Although considered rare, GAVE accounts for 4% of all non-variceal upper GIT bleeding presenting with either acute onset heavy GIT bleeding or occult GIT bleeding leading to chronic iron deficiency and/or anaemia both of which can be associated with significant morbidity and mortality [12]. In almost all cases, GAVE is associated with an underlying chronic medical condition [12]. In 30% of GAVE patients, cirrhosis of the liver is present whilst in non-cirrhotic GAVE patients, over 60% have an underlying autoimmune connective tissue disease most commonly SSc [12, 13]. Despite this strong association between autoimmune conditions and GAVE, very little is known about the epidemiology and aetiopathogenesis of this manifestation in SSc. To date, studies have indicated varying prevalences of GAVE depending on the indication for endoscopy, ranging from 0.6% in a Brazilian SSc cohort undergoing upper gastrointestinal endoscopy for investigation of a GI bleed, anaemia or GIT dysmotility (SSc cohort = 664, GAVE diagnosed *n* = 4) [14]; 1% in the European League Against Rheumatism Scleroderma Trials and Research (EUSTAR) network (SSc cohort *n* = 4090, GAVE diagnosed *n* = 49) [15]; 5.7% in SSc patients presenting with symptomatic anaemia (*n* = 264, GAVE diagnosed *n* = 15) [16]; and 22.5% in asymptomatic early diffuse SSc (dcSSc) patients who underwent endoscopy for another purpose as part of the Scleroderma: Cyclophosphamide Or Transplant (SCOT) trial (SSc cohort *n* = 103, GAVE diagnosed *n* = 23) [17, 18]. The real prevalence of GAVE is unknown and likely underestimated as we do not routinely perform endoscopy in asymptomatic SSc patients.

Although GAVE is recognised as occurring with an increased frequency in SSc, little is known also about its disease associations and impact on survival. Previous studies have been retrospective and mostly performed in small SSc patient cohorts with conflicting disease associations and outcomes. Therefore, our study sought to examine the epidemiology of GAVE in a large prospective Australian SSc cohort and to determine its disease associations and impact on survival.

## Methods

Consecutive SSc patients prospectively enrolled in the Australian Scleroderma Cohort Study (ASCS), a multi-centre study of risk and prognostic factors for clinically important outcomes in SSc, were included. The ASCS database collects comprehensive demographic and disease-related data on an annual basis. Written informed consent from all patients and ethical approval from all participating hospitals were obtained.

### Inclusion and exclusion criteria

We included all adult (>18 years) SSc patients recruited in the ASCS since January 2008 (cohort inception). All patients fulfilled the American College of Rheumatology / European League Against Rheumatism Classification criteria for SSc [19].

#### ASCS clinical data

SSc disease onset was defined as the first non-Raynaud’s phenomenon SSc disease clinical manifestation. Clinical manifestations and autoantibody status were defined as present if ever reported from the time of SSc diagnosis. Indications for upper gastrointestinal endoscopy included the following: (i) unexplained iron deficiency anaemia (Hb<120 g/dL); (ii) occult and/ or acute GI bleeding; (iii) delayed gastric emptying on nuclear transit studies or gastroesophageal reflux disease (GORD) unresponsive to treatment and/ or (iv) dysphagia or suspected oesophageal stricture. Occult GI bleeding defined as the presence of iron deficiency anaemia and/ or a positive faecal occult blood test in the absence of visible GI blood loss. Persistent GORD symptoms despite high-dose proton pump inhibitor therapy were considered unresponsive to therapy. GAVE (“watermelon stomach”) was diagnosed by its characteristic endoscopic appearance of rough parallel folds and dilated blood vessels departing from the pylorus and converging in the gastric antrum [6]. Scleroderma renal crisis (SRC) was defined as a combination of any two of the following three criteria (i) new onset severe hypertension (≥180 mmHg systolic and/or ≥100 mmHg) without an alternate aetiology; (ii) microangiopathic haemolytic anaemia; or (iii) rising creatinine. Digital ulcer (DU) was defined clinically by the treating physician as a visually discernible depth and a loss of continuity of epithelial coverage on a digit [20]. Interstitial lung disease (ILD) was defined as present by characteristic fibrotic changes on high-resolution computed tomography (HRCT) lung [19]. Pulmonary arterial hypertension (PAH) was defined as present if diagnosed by right heart catheterization according to international criteria [21]. Medication use data, prescribed at the discretion of the treating physician(s), and health-related quality of life data (HRQoL), measured using Medical Outcome Short Form-36 (SF-36) (a validated instrument for measuring HRQoL in SSc [22]), at GAVE diagnosis and during follow-up were recorded at each visit. In the SF-36, a score between 0 and 100 is calculated for both the physical component score (PCS) and the mental component score (MCS) which are standardized to normative population HRQoL scores. A score below 50 indicates a worse HRQoL than the background population with one standard deviation represented by 10 points. Patient status (alive or dead) was censored in June 2021.

#### Statistical analysis

Data are presented as mean ± standard deviation (SD) for normally distributed and median (25th–75th) for non-normally distributed continuous variables, and as number (percentage) for categorical variables. Differences in frequency were tested using chi-square and Fisher’s exact tests. Univariable and multivariable logistic regression were used to determine the associations of GAVE with clinical manifestations and serological parameters. Kaplan-Meier (K-M) survival curves were used to estimate survival in patients with and without GAVE. To estimate HRQoL, the patients’ PCS and MCS median scores from enrolment to last follow-up were calculated. Variables with a *p*-value <0.05 in univariable regression or variables deemed to be of clinical significance to the outcome with a *p*-value <0.10 were included in the multivariable logistic regression analysis. A two-tailed *p*-value of 0.05 or less was considered statistically significant. All statistical analyses were performed using STATA 15.1 (StataCorp LP, College Station, TX, USA).

## Results

### Patient characteristics

Our cohort consisted of 2039 SSc patients, of whom 216 (10.6%) had been diagnosed with GAVE over a median follow-up of 4.3 (1.7–8.4) years. Those with a history of GAVE compared with those without a history of GAVE were more frequently ANA positive (98.6% vs 94.9%, *p* = 0.019) with a speckled rather than a homogenous pattern (39.7% vs 29.3%, *p* = 0.003 and 9.6% vs 22.2%, *p* < 0.001 respectively), negative for antitopoisomerase-1 (Scl-70), U1 small nuclear ribonucleoprotein (U1RNP), and Scl/PM antibodies (4.0% vs 16.1%, *p* < 0.001, 3.5% vs 7.4%, *p* = 0.041, and 0.0% vs 2.0%, *p* = 0.042 respectively) and positive for anti-RNA polymerase (RNAP) III antibody (24.9% vs 8.3%, *p* < 0.001). Moreover, those with GAVE were more likely to have a history of anaemia (38.0% vs 15.2%, *p* < 0.001) and a history of a significant haemoglobin drop below normal (defined as >10 g/L) between clinical visits (53.8% vs 44.3%, *p* = 0.019) than those without a history of GAVE. There was no difference in acute phase reactants between these groups including inflammatory markers (CRP, ESR) nor in platelet count and albumin levels (Table 1). In terms of clinical manifestations, SSc patients with a history of GAVE compared with those without GAVE were more likely to experience other SSc vascular manifestations including telangiectasia (93.0 vs 85.1%, *p* = 0.002), calcinosis (48.8% vs 37.6%, *p* < 0.001), SRC (8.3% vs 3.1%, *p* < 0.001), and digital ulcerations, pits and amputations (54.2% vs 41.0%, *p* < 0.001, 67.0% vs 58.0%, *p* = 0.012 and 19.1% vs 12.4%, *p* = 0.006 respectively). Interestingly, there was no association between the presence of GAVE and the SSc-related cardiopulmonary manifestations, namely PAH and ILD (Table 1). Other clinical manifestations occurring more frequently in those with GAVE included GIT involvement comprising GORD, oesphageal and intestinal dysmotility (100% vs 92.4%, *p* < 0.001; 49.5% vs 38.7%, *p* = 0.002; and 38.4% vs 23.4%, *p* < 0.001, respectively) in addition to the co-morbidities of ischemic heart disease (IHD) and peripheral vascular disease (PVD) (17.8% vs 10.0%, *p* = 0.001 and 11.3% vs 6.9%, *p* = 0.044 respectively). Although the presence of GAVE was associated with a trend towards increased incidence of overall malignancy, this did not reach statistical significance (26.4% vs 20.7%, *p* = 0.055) (Table 1). Furthermore, those with a history of GAVE had more hospitalisations following GAVE diagnosis compared to those without a history of GAVE from study enrollment (63.4% vs 51.6%, *p* = 0.001). At date of censorship, fewer SSc patients with a history of GAVE than those without a history of GAVE were alive (79.2% vs 84.4%, *p* = 0.048). In terms of HRQoL, the presence of GAVE was associated with a significant reduction in the MCS of the SF-36 compared with those without a history of GAVE (39.7 (29.6–49.2) vs 43.6 (33.5–52.5), *p* = 0.002), which is a twofold higher difference than the minimally important difference [23] (Table 1).

**Table 1:** Patient characteristics by the presence of gastric antral vascular ectasia (GAVE)

Patient characteristics	GAVE (n=216) n (%) or median (IQR 25^**th**^-75^**th**^)	No GAVE (n=1823) n (%) or median (IQR 25^**th**^-75^**th**^)	***p***-value
Demographics			
Age at SSc disease onset*, years	49.51 (40.04-58.21)	46.70 (35.97-56.70)	0.051
Female	188 (87.0%)	1553 (85.5%)	0.535
Caucasian ethnicity	183 (91.5%)	1522 (90.9%)	0.768
Diffuse disease subtype	72 (35.3%)	401 (24.1%)	0.001
Follow-up, years	4.27 (1.72-8.39)	3.39 (1.00-7.25)	0.003
Autoantibodies**			
ANA positive	204 (98.6%)	1615 (94.9%)	0.019
ANA Pattern			
Centromere	99 (49.3%)	833 (52.4%)	0.397
Speckled	79 (39.7%)	461 (29.3%)	0.003
Nucleolar	47 (23.5%)	387 (24.6%)	0.725
Homogenous	19 (9.6%)	346 (22.2%)	<0.001
ENA subtype positivity			
Anti-Scl70	8 (4.0%)	264 (16.1%)	<0.001
Scl/PM	0 (0.0%)	33 (2.0%)	0.042
U1RNP	7 (3.5%)	121 (7.4%)	0.041
RNA Polymerase III positive	46 (24.9%)	129 (8.3%)	<0.001
Anaemia	79 (38.0%)	258 (15.2%)	<0.001
Drop in Hb of >10g/l between visits	91 (53.8%)	561 (44.3%)	0.019
Clinical manifestations**			
Digital ulcers	117 (54.2%)	736 (41.0%)	<0.001
Digital pitting	142 (67.0%)	1013 (58.0%)	0.012
Digital amputation	41 (19.1%)	221 (12.4%)	0.006
Telangiectasia	198 (93.0%)	1486 (85.1%)	0.002
Calcinosis	105 (48.8%)	649 (37.6%)	<0.001
GORD	216 (100.0%)	1659 (92.4%)	<0.001
GIT dysmotility			
Oesphageal	107 (49.5%)	706 (38.7%)	0.002
Bowel	83 (38.4%)	427 (23.4%)	<0.001
SSc Renal Crisis	18 (8.3%)	56 (3.1%)	<0.001
ILD	53 (58.2%)	477 (64.8%)	0.218
PAH^#^	24 (11.1%)	171 (9.4%)	0.413
Co-morbidities			
Smoking history (current or ever)	121 (56.8%)	885 (49.9%)	0.056
Ischemic heart disease	38 (17.8%)	175 (10.0%)	0.001
Peripheral vascular disease	18 (11.3%)	82 (6.9%)	0.044
Concurrent cancer diagnosis	57 (26.4%)	378 (20.7%)	0.055
Hospitalisations***	130 (63.4%)	899 (51.6%)	0.001
Medication			
Protein Pump Inhibitor	207 (95.8%)	1423 (78.1%)	<0.001
Histamine 2 receptor antagonist	73 (33.8%)	376 (20.6%)	<0.001
Anticoagulant medication	15 (6.9%)	80 (4.4%)	0.092
Antiplatelet agent	69 (31.9%)	534 (29.3%)	0.419
Cyclophosphamide	20 (9.3%)	150 (8.2%)	0.604
HRQoL^##^			
Physical component score (PCS)	53.07 (42.41-58.55)	53.56 (44.49-58.70)	0.731
Mental component score (MCS)	39.73 (29.58-49.19)	43.56 (33.48-52.49)	0.002

*Abbreviations*: *SSc* systemic sclerosis, *GORD* gastroesophageal reflux, *PAH* pulmonary arterial hypertension, *ILD* interstitial lung disease, *GIT* gastrointestinal tract, *ACA* anticentromere, *Scl-70* antitopoisomerase-1, *U1RNP* U1 small nuclear ribonucleoprotein, *RNAP* anti RNA Polymerase, III, *SD* standard deviation, *HRQoL* health related quality of life

*SSc onset defined as the first non-RP disease manifestations symptom of SSc (Raynaud phenomenon or other) *disease duration defined as from first non-Raynaud’s disease manifestation,

**autoantibody and clinical manifestations defined as present if ever present from SSc diagnosis

***hospitalisations defined as ever admitted to hospital from ASCS enrollment in those without GAVE and in those with GAVE hospitalisations were defined as since GAVE diagnosis

^#^PAH diagnosed on right heart catheterization (RHC) according to international criteria [11]

^##^HRQoL was defined using the SF-36 study short form which provides a score range from 0-100. Scores below 50 indicate worse HRQoL than the population normative score and every 10 points indicates 1 standard deviation. These scores can be summarized into the physical component score (PCS) and mental component score (MCS), Scores below 50 indicate worse HRQoL than the population normative score and every 10 points indicates 1 standard deviation. The HRQoL score was calculated based on mean HRQoL over the follow-up period in both those with and without GAVE.

### Patient characteristics of those with GAVE (Table 2)

In our cohort, the majority (92.3%) of SSc patients with GAVE were diagnosed with GAVE following their SSc diagnosis; however, in 7.7% of patients, GAVE was the first clinical manifestation of SSc. Overall, GAVE patients were predominantly Caucasian (91.5%) females (87.0%) with limited SSc disease subtype (lcSSc) (64.7%) at a median age of 55.9 (47.3–66.5) years and SSc disease duration of 4 (0.8–12.4) years at GAVE diagnosis. GAVE occurred early in SSc disease course (within the first 5years), with dcSSc patients more likely to be diagnosed with GAVE earlier in their SSc disease course (3.1 vs 5.3 years, *p* = 0.003) compared with lcSSc. The majority of GAVE patients were positive for antinuclear antibody (ANA) (98.6%), the most common pattern being centromere (49.3%), followed by speckled (39.7%), nucleolar (23.5%) and homogenous (9.6%); and positive for RNAP III (32.9%) but negative for Scl-70; U1RNP; and PM/Scl (96.0%, 96.5% and 100% respectively). At GAVE diagnosis, 34.2% of SSc patients had a preceding acute haemoglobin (Hb) drop of 10 g/L and 48% reported an increased sensation of breathlessness whilst 42.3% had a preceding decline in their diffusing capacity of carbon dioxide (DLCO) on RFTs. In terms of GAVE treatment, the majority received a PPI (95.8%), whilst a third (33.8%) received a H2RA and just over a quarter of patients (25.5%) received endoscopic laser therapy (Table 3).

**Table 2:** Patient characteristics in those with GAVE at GAVE diagnosis (n=216)

Characteristic	GAVE n (%) or median (IQR 25^**th**^-75^**th**^)	***p***-value
Demographics		
Age at GAVE diagnosis, years	55.94 (47.25-66.46)	
SSc duration^a^ at GAVE diagnosis, years	4.00 (0.84-12.42)	
Limited disease subtype	5.25 (0.50-16.08)	
Diffuse disease subtype	3.08 (1.25-9.59)	0.003
Female	188 (87.0%)	
Diffuse disease subtype	72 (35.3%)	
Caucasian	183 (91.5%)	
Follow-up from GAVE diagnosis, years	6.88 (2.99-11.16)	
Serological markers at GAVE diagnosis		
inflammatory markers		
CRP	4.00 (2.75-7.50)	
ESR	17.50 (9.00-27.00)	
platelets	298.00 (256.00-351.00)	
albumin	37.00 (35.00-40.00)	
Anaemia at GAVE diagnosis	19 (31.7%)	
Drop in Hb of 10g pre-GAVE diagnosis	91 (53.8%)	
Drop in Hb of 10g post GAVE diagnosis	75 (37.5%)	
Increase in MRSS in preceding 12months of dx	15 (24.2%)	
Clinical markers at GAVE diagnosis		
Increasing breathlessness	46 (23.5%)	
Decrease in DLCO	71 (42.3%)	

*Abbreviations*: *SSc* systemic sclerosis, *GAVE* gastric antral vascular ectasia, *CRP* C-reactive protein, *ESR* erythrocyte sedimentation rate, *Hb* haemoglobin, *MRSS* modified rodnan skin score, *DLCO* diffusing capacity for carbon monoxide

^a^disease duration defined as from first non-Raynaud’s disease manifestation

**Table 3.** Determinants of GAVE on univariable analysis

Variable	Odds Ratio (95% CI)	***p***-value
Age at SSc onset^a^, years	1.01 (1.00-1.02)	0.099
Female	1.14 (0.75-1.73)	0.535
Diffuse disease subtype	1.72 (1.26-2.34)	0.001
DU, pits or amputation	1.67 (1.22 to 2.29)	0.001
GIT dysmotility	1.75 (1.31-2.33)	<0.001
Telangiectasia	1.52 (1.05-2.20)	0.027
Calcinosis	1.48 (1.12-1.97)	0.007
SSc renal crisis	2.87 (1.65-4.98)	<0.001
PAH	1.21 (0.77-1.90)	0.414
ILD	0.76 (0.49-1.18)	0.219
ANA centromere positivity	1.22 (0.91-1.63)	0.186
U1RNP	0.45 (0.21-0.99)	0.046
Scl-70 positivity	0.22 (0.11-0.44)	<0.001
RNAP III positivity	3.64 (2.49 to 5.31)	<0.001

*Abbreviations*: *SSc* systemic sclerosis, *GAVE* gastric antral vascular ectasia, *GIT* gastrointestinal tract, *DU* digital ulcers, *ANA* antinuclear antibody, *ACA* anticentromere, antitopoisomerase-1 (Scl-70), U1 small nuclear ribonucleoprotein (U1RNP), anti RNA Polymerase (RNAP) III, confidence interval (CI)

^a^age defined as from first non-Raynaud’s disease manifestation

### Survival analysis in those with and without GAVE (Fig. 2)

**Fig. 2:**
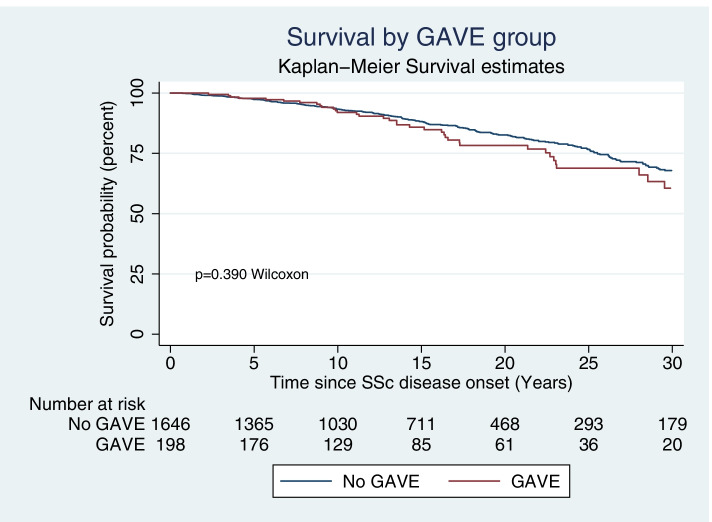
Kaplan-Meier curve for survival by GAVE status. This Kaplan-Meier survival curve for survival shows no significant difference in survival between those with and without GAVE (*p* = 0.39) in our SSc cohort. Abbreviations: systemic sclerosis (SSc) gastric antral vascular ectasia (GAVE). *Disease onset defined as from first non-Raynaud’s disease manifestation

There was no significant difference in survival between those with and without GAVE (*p* = 0.39), in our SSc cohort (Fig. 2). Of the 336 SSc patients (17.9%) who died during the follow-up period, the median time to death from SSc disease onset was 16.3 (9.8–25.5) years for those with GAVE and 14.7 (7.8–25.0) years for those without GAVE (*p* = 0.409). Time to death from GAVE diagnosis was 7.7 (3.5–11.3) years. In both those with and without GAVE, the leading cause of death was a consequence of their SSc disease manifestations (61.5% and 59.3%. *p* = 0.78 respectively).

### Determinants of GAVE (Tables 3 and 4)

Determinants of GAVE by univariable analysis are summarised in Table 3. Associations with GAVE include the presence of dcSSc, telangiectasia, calcinosis, SRC, DU, pits and amputation in addition to GIT dysmotility. The presence of RNAP III was associated with GAVE, whilst the presence of Scl 70 and ENA RNP were protective.

**Table 4:** Determinants of GAVE on multivariate analysis

Variable	Odds Ratio (95% CI)	***p***-value
**a) Determinants of GAVE**		
Age at SSc onset^a^, years	1.01 (1.00-1.02)	0.092
Diffuse disease subtype	1.48 (1.06-2.06)	0.023
GIT dysmotility	1.57 (1.15-2.14)	0.005
SSc renal crisis	2.10 (1.12-3.91)	0.020
DU, pits or amputation	1.46 (1.02-2.07)	0.036
**b) Determinants of GAVE by autoantibody status**		
Age at SSc onset^a^, years	1.01 (1.00-1.03)	0.015
ANA centromere positivity	1.28 (0.88-1.86)	0.204
Scl-70 positivity	0.26 (0.11-0.62)	0.002
RNAP III positivity	3.92 (2.50-6.16)	<0.001
GIT dysmotility	1.66 (1.19-2.32)	0.003
DU, pits or amputation	1.59 (1.10-2.30)	0.014

*Abbreviations*: *SSc* systemic sclerosis, *GAVE* gastric antral vascular ectasia, *GIT* gastrointestinal tract, *DU*, *Scl-70* antitopoisomerase-1, *RNAP* anti RNA Polymerase III, *CI* confidence interval

^a^age defined as from first non-Raynaud’s disease manifestation

Assessments of determinants of GAVE by multivariable regression analysis was performed in two separate analyses due to collinearity, one including disease subtype and the other including autoantibody profiles; both are summarised in Table 4. Determinants of GAVE in the disease subtype analysis included the presence of the dcSSc (OR 1.48, *p* = 0.02), presence of DU, pits or digital amputation (OR1.46, *p* = 0.04), GIT dysmotility (OR1.57, *p* = 0.01) and SRC (OR = 2.10, *p* = 0.02) (Table 4a). Determinants of GAVE by autoantibody status included the presence of RNAP III antibody (OR 3.92, *p* < 0.001), absence of Scl-70 antibody (OR 0.26, *p* = 0.001), presence of GIT dysmotility (OR 1.66, *p* = 0.003), the presence of DU, pits or digital amputation (OR 1.59, *p* = 0.014) and age at SSc onset (OR 1.01, *p* = 0.015) (Table 4b).

## Discussion

Our study is the largest Australian study and second largest international study, after the EUSTAR network study [15], describing the epidemiology, clinical characteristics, determinants and outcomes of GAVE in a large SSc cohort. In our cohort of 2039 SSc patients, 10.6% of SSc patients were diagnosed with GAVE over a median follow-up of 4.3 (1.7–8.4) years, which fits within the reported prevalence range in the literature (0.6–22.3%) [14–16, 18]. Consistent with the literature, SSc patients with GAVE in our study were older at SSc disease onset (49.2 (40.0–58.2) vs 46.7 (35.9–56.7) years, *p* = 0.05); more likely to have dcSSc (35.3% vs 24.1%, *p* < 0.001) [14]; more likely to be ANA and RNAP III antibody positive [15] and negative for Scl-70 [15, 18]. As reported previously [16], those with GAVE in our cohort were more likely to display SSc vascular manifestations including telangiectasia and SRC without an increased association with PAH. We also found an association between the presence of GAVE and calcinosis. Whether calcinosis represents a vascular manifestation of SSc is contentious. The pathogenesis of calcinosis in SSc is unknown with some literature to support its mechanism occurring as a consequence of local trauma, chronic inflammation, vascular hypoxia, and /or dysregulation of bone matrix proteins [24]. Contrary to the EUSTAR study [15], those with GAVE in our cohort compared with those without GAVE were more likely to have concurrent GIT manifestations including the presence of GORD and/or GIT dysmotility and the presence of DU, pits and digital amputation. The increased association of GAVE with a concurrent diagnosis of GIT manifestations including GORD maybe a selection bias as a diagnosis of GAVE in our cohort required upper gastrointestinal endoscopy. The majority of SSc patients with GAVE in our cohort were female (87%), similar to our whole ASCS cohort, with a median age at GAVE diagnosis of 55.9 (47.3–66.5) years similar to the EUSTAR cohort (90% female and 56 years respectively) [15]. SSc disease duration at GAVE diagnosis was shorter in those with dcSSc compared with lcSSc (3.1 vs 4.0 years, *p* = 0.003), a trend which was seen in the EUSTAR cohort but did not reach statistical significance (13 vs 19 months, *p* = 0.63) [15], indicating that GAVE is an early SSc disease manifestation (occurring within the first 5 years of disease onset), especially in those with dcSSc. The main determinants of GAVE in our SSc cohort when analysed by disease subtype included the presence of SRC, GIT dysmotility, DU, pits or amputations and dcSSc, whilst the presence of older age at SSc disease onset, GIT dysmotility, DU, pits or amputation, RNAP III positivity and Scl-70 negativity were the main determinants of GAVE when analysed by autoantibody status. Despite GAVE being associated with reduced HRQoL, our study indicates that its presence alone does not reduce survival.

Despite the increased frequency of GAVE in SSc, the pathophysiological mechanism of this association is not well understood and theories are based on small studies and case reports [25]. GAVE in SSc can be classified as either a gastric or vascular manifestation or a combination of both. Additionally, the histological hallmarks of GAVE including the presence of mucosal hyperplasia with capillary ectasia and thrombosis, fibromuscular hyperplasia of the lamina propria and abnormal vessels in the submucosa [8, 9] are similar to other vascular SSc manifestations such as telangiectasia, SRC and PAH [10, 11, 26]. Despite our study showing an association between GAVE and other SSc-related vascular manifestations, namely DU, pits, amputations, telangiectasia and SRC, it is interesting that there does not appear to be an association between the presence of GAVE and PAH in our SSc cohort or in the wider literature [14–16, 18], highlighting our relatively basic understanding of the pathophysiological mechanisms in SSc. This lack of association between GAVE and PAH may in part be due to an underappreciation of GAVE in the very unwell SSc-PAH cohort who are managed conservatively with iron and /or blood transfusions rather than investigated with endoscopy.

Although considered rare in the general population, the reported prevalence of GAVE in SSc cohorts range from 1 to 22% [27], with a prevalence of 10.6% in our SSc cohort, indicating that there is an underappreciation of the frequency of GAVE in SSc. It must be noted, however, that endoscopy was only preformed in our cohort when there was a subjective or objective finding consistent with SSc-GIT involvement (indications are outlined in the “Methods” section). As such, the prevalence of GAVE in our cohort is not generalizable to an asymptomatic SSc cohort without GIT manifestations whereby the prevalence may be much higher. This is nicely illustrated in the Scleroderma: Cyclophosphamide or Transplant (SCOT) trial [18], where the prevalence of GAVE was found to be 22%. In this trial, all early dcSSc patients underwent endoscopy regardless of GIT symptoms. The wide prevalence of GAVE (1–22%) reported in the literature may therefore be more related to the indication for endoscopy (asymptomatic versus symptomatic of GIT disease). Given GAVE is one of the few SSc manifestations that can be easily and relatively non-invasively diagnosed and treated, perhaps consideration should be given to screening for GAVE in certain at-risk SSc phenotypes with 6–12 monthly iron studies and upper gastrointestinal endoscopy in those that are iron deficient. Our study would indicate that there are two at-risk SSc phenotypes, both occurring early in their SSc disease course (within the first 5 years of SSc disease onset), defined based on either their disease subtype or autoantibody status. The first phenotype is early dcSSc with concurrent GIT dysmotility, DU, pits or amputations and presence of SRC, whilst the second phenotype is early SSc patients, regardless of disease subset, with concurrent RNAP III positivity, Scl-70 negativity, and presence of GIT dysmotility, DU, pits and/ or amputations. Furthermore, we should endeavour to refer all SSc patients with iron deficiency with or without anaemia for endoscopy. Given GAVE was the first clinical manifestation of SSc in 7.7% of our GAVE cohort, increased awareness and physician education should be directed at the importance of evaluating individuals diagnosed with GAVE in the absence of cirrhosis for an underlying autoimmune condition with an appropriate clinical history, examination, nailfold capillaroscopy and serological tests including antinuclear antibodies.

In terms of GAVE treatment, SSc patients in our cohort were treated in accordance with expert recommendations [28] including PPI and / or H2RA therapy (95.8% and 33.8% respectively), with endoscopic treatment and surgery being reserved for those with refractory severe GAVE (with laser therapy being performed on just over a quarter of patients (25.5%) in our cohort) [28]. Despite small studies and case series showing a therapeutic effect of cyclophosphamide on GAVE when used for other indications such as ILD or progressive skin disease, our data did not show a benefit with immunosuppressive therapy including cyclophosphamide [29, 30]. However, this study did not specifically address the exact indication for cyclophosphamide; its therapeutic duration and /or therapeutic response so we cannot draw any conclusions as to the benefit of cyclophosphamide therapy in management of SSc GAVE.

With regard to quality of life, our SSc cohort reported low HRQoL which is consistent with other SSc cohorts [31, 32], which in our study was further negatively impacted by the presence of GAVE compared with those without GAVE (39.7 vs 43.6, *p* = 0.002). The presence of GAVE in our cohort was strongly associated with GIT dysmotility and GIT involvement which is in itself a well-recognised significant contributor to SSc-related morbidity and reduced HRQoL [2–4]. Furthermore, those with GAVE compared to those without GAVE had a significantly higher number of hospitalisations during their follow-up period (63.4% vs 51.6%, *p* = 0.001) highlighting the unpredictable nature of SSc disease course, which in other chronic diseases has been shown to negatively impact on patient-reported HRQoL [33, 34]. These further highlights that improving HRQoL is an area of unmet need in SSc, which requires a more targeted understanding before significant improvements can be made.

Strengths of our study include its well-characterized SSc cohort followed prospectively over a substantial period of time in addition to clearly defined and recorded clinical manifestations and survival data. Although endoscopy was performed by clinical indication as described in the “Methods” section, the exact indication for each endoscopy was not recorded. Also, we collect data on the presence or absence of GAVE in our cohort rather than those who do and do not undergo endoscopy. Thus categorisation by gastroscopy (yes/ no) and or/ indication for endoscopy was not feasible and we cannot exclude the potential for selection bias. In addition, as GAVE may be mild and not detected clinically, we believe that the true prevalence of GAVE in our cohort is likely to be an underestimate as iron levels are not available to identify those with early blood loss.

## Conclusions

GAVE is an under-recognised SSc disease manifestation, which can be easily diagnosed and treated, occurring with a prevalence of 10.6% in our SSc cohort. Increasing physician awareness as to the presentation and complications of GAVE is an important step in increasing recognition of this disease entity. Furthermore, identifying at-risk SSc phenotypes, as this study has done, raises the important question as to the benefits of targeted screening of these high-risk phenotypes in the hopes of reducing GAVE-related morbidity and improving HRQoL.

## Data Availability

The dataset supporting the conclusions of this article can be made available on request through the corresponding author.

## Abbreviations

ANA: Antinuclear antibody
ASCS: Australian Scleroderma Cohort Study
DU: Digital ulcers
GAVE: Gastric antral vascular ectasia
GORD: Gastroesophageal reflux disease
H2RA: Histamine type 2 receptor antagonists
HRQoL: Health-related qualify of life
IHD: Ischemic heart disease
lcSSc: Limited SSc disease subtype
RNAP III: RNA Polymerase III
SSc: Systemic sclerosis or scleroderma
SRC: SSc renal crisis
PAH: Pulmonary arterial hypertension
PVD: Peripheral vascular disease
PPI: Proton pump inhibitor
